# 3D assessment of mandibular skeletal effects produced by the Herbst appliance

**DOI:** 10.1186/s12903-020-01108-4

**Published:** 2020-04-16

**Authors:** Yi Fan, Paul Schneider, Harold Matthews, Wilbur Eugene Roberts, Tianmin Xu, Robert Wei, Peter Claes, John Clement, Nicky Kilpatrick, Anthony Penington

**Affiliations:** 1 Department of Orthodontics, Peking University School and Hospital of Stomatology, Beijing, 10081 China; 2 Melbourne Dental School, University of Melbourne, Melbourne, 3053 Australia; 3 Facial Science, Murdoch Children’s Research Institute, Melbourne, 3052 Australia; 4 Department of Human Genetics, 3000 Leuven, KU Belgium; 5 Medical Imaging Research Centre, Universitair Ziekenhuis, 3000 Leuven, Belgium; 6 Department of Orthodontics and Oral Facial Genetics, Indiana University-Purdue University Indianapolis, Indianapolis, 46236 USA; 7 Department of Electrical Engineering, KU Leuven, Leuven, 3000 Belgium; 8 The University of Melbourne Department of Paediatrics at the Royal Children’s Hospital, 50 Flemington Rd, Parkville VIC, Melbourne, 3052 Australia

**Keywords:** Herbst appliance, Class II malocclusion, Geometric morphometrics

## Abstract

**Background:**

A functional appliance is commonly used to optimize the development of the facial skeleton in the treatment of Class II malocclusion. Recent three-dimensional(3D) image-based analysis offers numerous advantages in quantitative measurement and visualization in orthodontics. The aim of this study was to localize in 3D the skeletal effect produced by the Herbst appliance on the mandible using the geometric morphometric technique.

**Methods:**

Twenty patients treated with a Herbst appliance and subsequent fixed appliances were included. Cone-beam computed tomography (CBCT) images were taken before treatment (T1), 8 weeks after Herbst appliance removal (T2), and after subsequent fixed appliance treatment (T3). Spatially dense morphometric techniques were used to establish the corresponding points of the mandible. The mandibular morphological changes from T1-T2, T2-T3, and T1-T3 were calculated for each patient by superimposing two mandibular models at two time points with robust Procrustes superimposition. These changes were then compared to the morphological changes estimated from normative mandibular growth curves over the same period. The proportion of cases exceeding the growth expression for controls was compared to a normal population using a one tailed binomial test.

**Results:**

Approximately 1.5–2 mm greater condylar changes and 0.5 mm greater changes in the chin occurred from Tl to T2. This effect lasted until the completion of treatment (T1-T3), but there was no obvious skeletal effect during the orthodontic phase (T2-T3). Approximately 40–50% of the patient sample exceeded condylar growth by > 1.5 mm compared to untreated controls (*p* < .05). However, changes at the chin were not statistically significant.

**Conclusions:**

The principal skeletal effect of Herbst appliance treatment was additional increase in condylar length for about half of the sample. This inconsistency may relate to the degree of mandibular growth suppression associated with a specific malocclusion.

## Background

In the treatment of Class II malocclusion, an early phase functional appliance is commonly used for the correction of sagittal jaw discrepancies and to optimize the development of the facial skeleton [1, 2]. The classic removable orthodontic appliances require patient compliance so many practitioners prefer fixed functional options, such as the Herbst appliance. The Herbst appliance rigidly connects the first maxillary molar with the lower dentition on both sides through a telescopic (rod and tube) mechanism, thus keeping the mandible in a continuous anterior position. Therapy typically lasts 6 to 9 months [3]. The condyles are positioned inferiorly and anteriorly relative to the original condyle-fossa position. As a result, mandibular jaw and muscle function may result in growth enhancement to correct the skeletal malocclusion [4].

Although many clinicians agree that early Herbst appliance treatment is useful for correcting a Class II relationship [5], the nature of the orthopedic effect on the form of the mandible compared to normal growth remains controversial. When evaluating the clinical response for growing children, it is difficult to separate an orthopedic effect from normal growth. Animal experiments are not necessarily applicable to humans [6, 7]. For a clinical investigation, obtaining an identical control group is challenging because it is difficult to match the magnitude of skeletal discrepancy, dental malocclusion, age, maturation, and follow-up evaluation periods, especially in retrospective clinical studies [5].

Another difficulty in interpreting the evidence has been related to inconsistencies and fundamental limitations in the techniques available for measuring treatment outcomes. Lateral cephalometric studies in 2D evaluate mandibular morphology as a profile image. Inter-landmark distances, such as mandibular length (Condylion-Gnathion or Co-Pogonion), corpus length (Gonion-Gnathion, Gonion-Menton or Gonion- Pogonion) and ramus height (Condylion-Gonion) are adversely affected by projection errors, deviations in patient positioning and overlay of the structures on the left and right sides of the mandible [8]. While some studies have used cephalometric analysis to show an additional mandibular length increment in the 2 to 3 mm range [5, 9], other studies demonstrate minimal orthopedic effects on the mandible [10]. This difference may be attributed to the difficulty in reliably recognizing the condylion point on 2D radiographs [11]. Although improved landmark recognition is achieved by rotating the reconstructed craniofacial structures in 3D with CT or CBCT images, the choice of landmarks and the planes of measurement are problematic. Selection of some measurements and exclusion of others can lead to biased results because it does not necessarily represent the overall shape of the mandible in 3D.

Geometric morphometrics, the multivariate statistical analysis of shape or form, includes methods to analyze spatially dense landmark coordinates [12]. In contrast to conventional methods, which analyze subsets of derived linear distances and angle measurements, the whole surface of the object is analyzed and compared. Although these technologies have been adopted widely in biology, the full potential of this method has not been exploited in dentistry. There is huge potential with emerging 3D imaging technologies to clarify the true orthopedic effect of functional appliances on the form of the mandible.

The purpose of this study is to assess the orthopedic effect of the Herbst appliance on the mandible in 3D using geometric morphometrics. This is achieved by comparing changes following Herbst appliance treatment to the morphological changes estimated from normative mandibular growth curves [13].

## Methods

### Sample

The cohort consisted of 20 patients treated in two phases with a Herbst appliance and subsequent fixed appliances (6 males, 14 females; mean age ± SD: 12.76 ± 0.89 years). Their records were sourced retrospectively from an orthodontic clinic near Melbourne, Australia. The pre-treatment inclusion criteria were: [1] Class II skeletal (ANB > 4 degrees), [2] bilateral Class II molar relationships > 4 mm, [3] intact permanent dentition, and [4] Phase 1 Herbst appliance treatment started near peak pubertal growth, which was defined as cervical vertebral maturation assessment (CVM) stage 3–4 [14]. Patients with other craniofacial anomalies or history of previous orthodontic treatment were excluded.

**Fig. 1:**
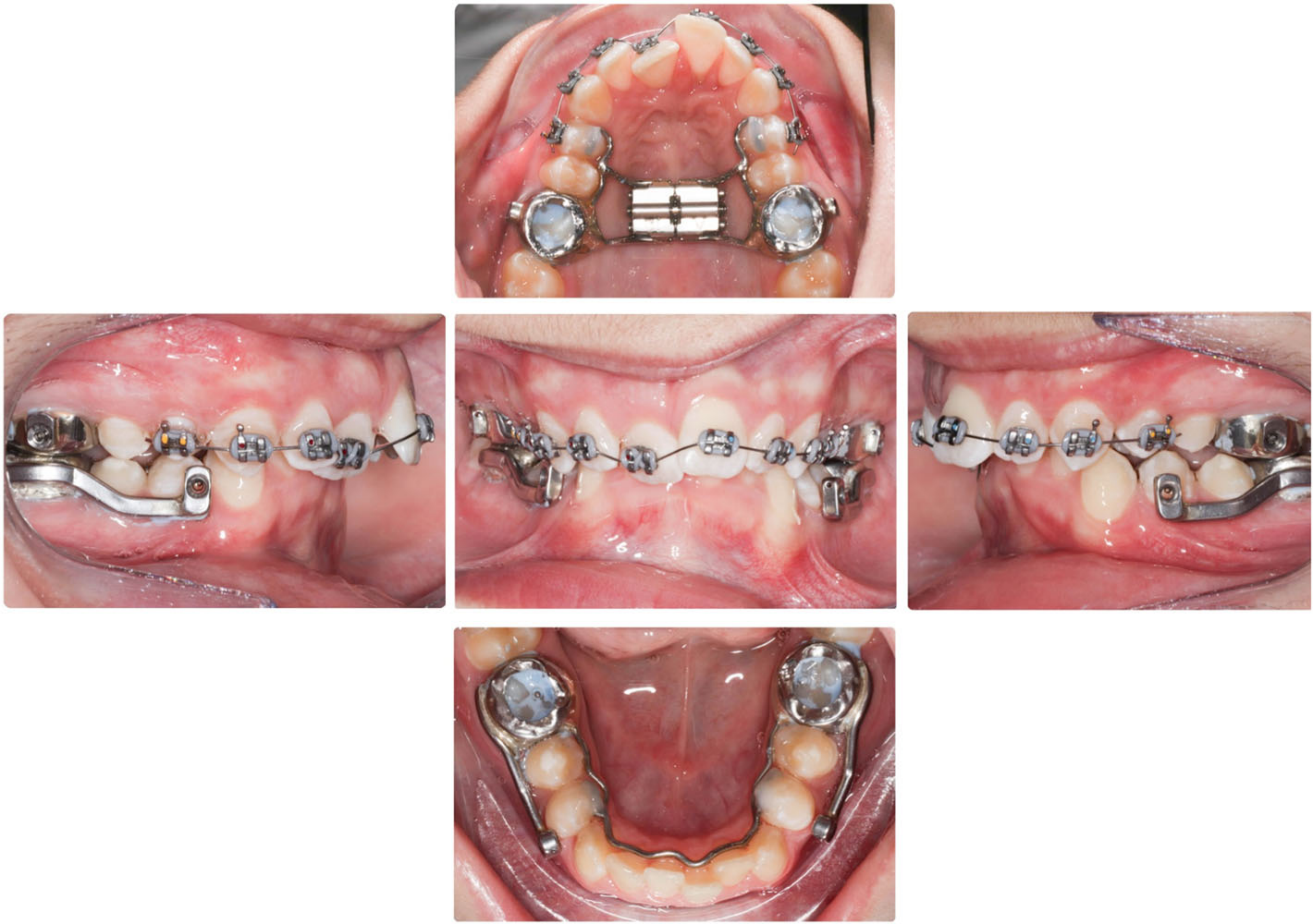
Intraoral photos of the cantilever Herbst appliance. The Herbst consists of four stainless steel crowns covering the four first molars. A cantilevered arm extended forwards from the mandibular first molar to the level of the mandibular first premolar

The Herbst appliance consisted of stainless-steel crowns fitted to the maxillary and mandibular first permanent molars and a cantilevered arm extended forwards from the mandibular first molar to the level of the mandibular first premolar. A Hyrax expansion screw appliance connected the maxillary first molars and a 0.040-in. diameter stainless steel lingual arch was used to connect the mandibular first molars (Fig. 1). The mandible was initially advanced 5 mm with subsequent 2 mm advancements to achieve an over-corrected edge-to-edge incisal position. The mean treatment time for the orthopedic phase with the Herbst appliance was 7.79 ± 1.82 months, and the fixed orthodontic phase was 22.08 ± 3.69 months.

The morphological changes due to treatment for each patient were determined by comparing the growth observed for the patient to a model for normal mandibular growth, which consisted of population-based, healthy pre-treatment orthodontic patients with a range of occlusal classifications [13]. These data were derived from a sample of 782 subjects (268 males and 386 females) of predominantly European descent that was based on a cross-sectional mandibular normal growth study (8.5–19.5 years) conducted in Melbourne, Australia. Subjects with a history of craniofacial anomalies, trauma or multiple missing teeth were excluded. Statistical outliers were excluded as previously described [13]. In brief, the study subjects and normal controls were drawn from the same general area. The evaluation interval (T1–3) for each patient was compared to the relevant portion of the normal growth curve based on the age and sex of the patient.

### Images

The CBCT images at T1–3 were prescribed by specialist orthodontists in private practice as part of their routine clinical protocol. For the Herbst group, the images were taken at pre-treatment (T1), 8 weeks after completion of the Herbst phase (T2), and after the fixed appliances were removed (T3). All the patients used to establish the normal growth curve had CBCT images exposed before and after orthodontic treatment as part of the usual standard of care. All patients in the control and experimental groups were instructed to bite in maximum intercuspation during scanning. Ethical approval to access the images retrospectively was obtained from the University of Melbourne Human Research Ethics Committee (ID: 1647544.1 and 1,647,867) and written informed consent was obtained from each participant’s guardian/s for inclusion in the study.

### Mandible segmentation

The mandible was automatically segmented from each CBCT image of the head using a marker-based watershed transform as previously described [15]. The outer surface for each mandible was represented by a cloud of dense points, linked into a ‘mesh’ of the surface. This was created by running the marching cubes algorithm in MATLAB on the segmented mandibular volume.

### Template mapping

Spatially-dense morphometric techniques cover the mandibular surface with a large number of points that capture the morphology of the entire mandible, including areas like the condyles and chin where traditional anatomical landmarks are poorly defined by local geometric features. An automatic template mapping strategy was used to ensure that each of the 17,415 defined points on one mandible corresponds with an anatomically similar point on the others [13, 16]. This procedure ensures that the morphological changes were measured over the same points between different patients. An open-source implementation of the mesh-to-mesh mapping algorithm is available at https://github.com/TheWebMonks/meshmonk [17].

### Quantifying the orthopedic effect

The overall morphological changes of the mandible from T1-T2, T2-T3, T1-T3 were calculated for each patient in the Herbst group. This was achieved by superimposing the mandibular models at each evaluation interval with robust Procrustes superimposition [16]. It mathematically translates and rotates one object so that it is aligned as closely as possible with the other by minimizing the sum of the overall difference between the objects [18]. In the case of two consecutive mandibular images, any remaining difference between the two images after alignment represents the morphological changes during the observation period. A ‘robust Procrustes’ superimposition, gives greater weight in the alignment process to regions of the two shapes which are most similar to each other [19]. Essentially this automatically estimates regions that change the least, thus provides an automatic and potentially reliable strategy for superimposition of the mandible in 3D that does not rely on pre-defined stable regions. This method will take the three-dimensional changes (sagittal, vertical and transverse) into account. The mandibles were iteratively aligned by estimating and applying: 1) the weighted Procrustes rotation and translation of each mandible onto the template, and 2) adjusting the ‘weights’ according to influence on transformation estimated by the next iteration to those points that were closest between the mandibles [13]. A weight of zero was imposed on the landmarks representing the teeth to eliminate the effect of teeth during mandibular superimposition. The total morphological changes were calculated at each corresponding point and visualized using colormaps projected onto the mandibular template. Although the teeth had no influence on the superimposition, they were included in the visualization of the dental alveolar effect because that is of primary interest to orthodontists.

Morphological changes observed were a combination of the orthopedic effect and natural growth. The growth expected by each patient during the interval of treatment was estimated on the growth curve according to age and sex using kernel regression [13]. This approach captured the non-linear mandibular morphological changes at each point of the mandible during adolescence. Sex-specific expected mandibles at the two ages were synthesized using this model. The orthopedic change associated with treatment was determined by subtracting the growth estimate from the overall morphologic change experienced by the patient.

**Fig. 2:**
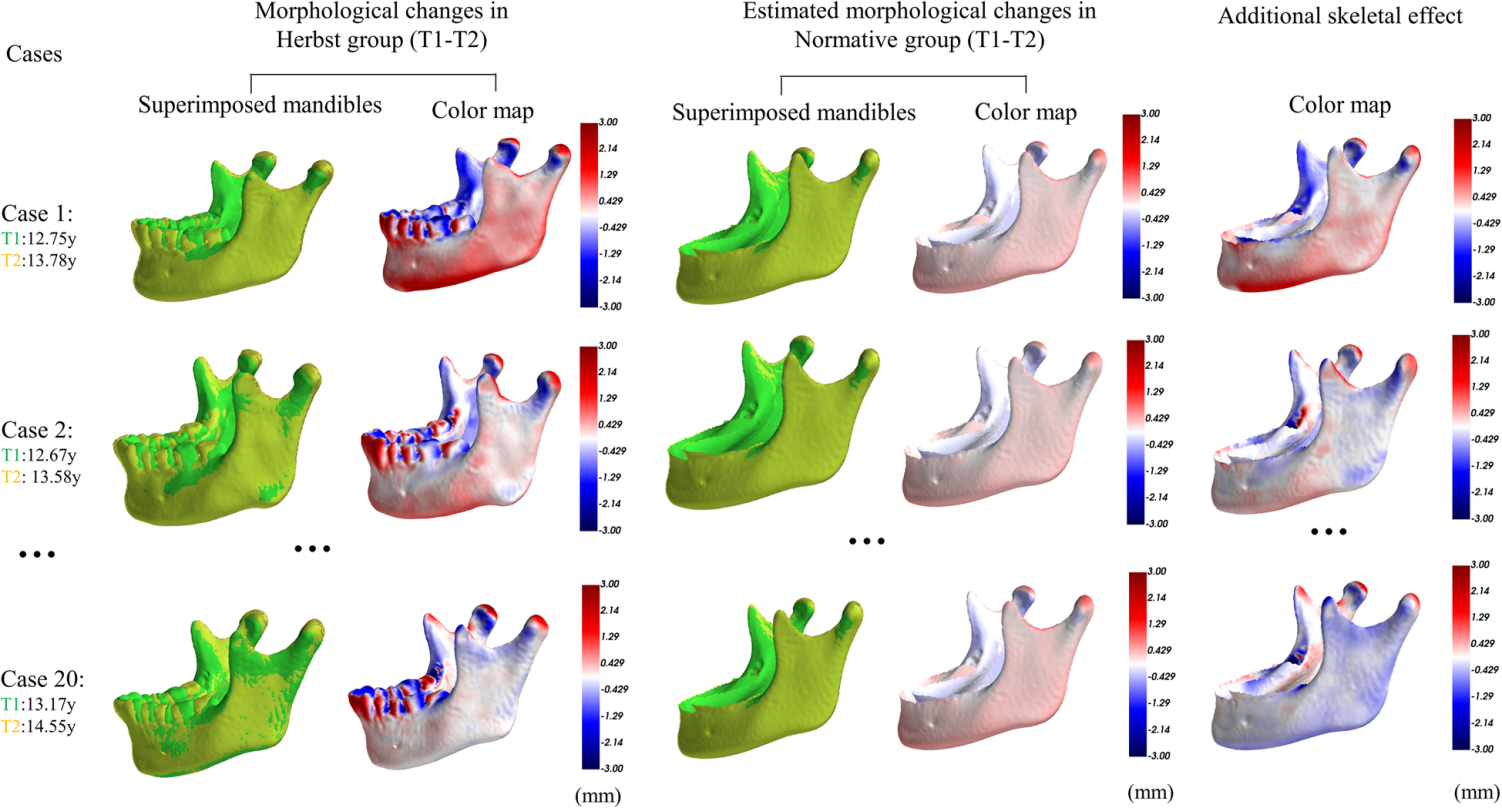
Quantification of the additional skeletal effect produced by the Herbst appliance. The morphological changes for each case in the Herbst group are shown in the first column; T2 image (yellow) is superimposed on T1 image (green). The adjacent color map shows the morphological changes that occurred in this interval, with red indicating regions of outward changes, white zero changes and blue inward changes. For example, outward changes occur at the condylar head and inward changes at the condylar neck in case one. The molars move mesially and the lower incisors procline anteriorly. Estimated morphological changes of the corresponding age- and sex-matched mandible during the same period is shown in column 2. Column 3 subtracts the morphological changes in the first and second column, indicating the additional skeletal effects for each case and these are used to calculate the mean morphological changes for the Herbst group

The orthopedic effect due to Herbst treatment (T1-T2) was measured individually for each patient (Fig. 2). The mean was calculated and compared to the expected morphologic change due to natural growth. After subtracting the growth effect, the additional mandibular morphologic change for each patient and the group effect were illustrated with color maps projected onto the template mandible. The additional skeletal (orthopedic) effect during the orthodontic phase (T2-T3) as well as the overall treatment period (T1-T3) were calculated in the same way. All the analyses were performed using custom-written code in the Python programming language.

### Statistical test

Without matched longitudinal images of an appropriate control group we cannot calculate the variation in normal growth rates on which to base a statistical inference of the difference in growth rates between the patient group and the normal population. However, an estimate of significance can be obtained by assuming growth rates in the population are distributed symmetrically around the central tendency of the distribution of growth rates. In other words, the proportion of individuals in the normal population growing faster than predicted by the model of normal growth and the proportion of individuals growing more slowly are assumed to be equal. The proportion of individuals in the Herbst group growing faster or slower than predicted is then compared to this assumed normal pattern using a one-tailed binomial test. Specifically, for each point on the mandible for each individual in the Herbst group we calculate whether the change is more or less than predicted from the model. The *P* value was generated for each point on the mandible and was plotted in a bicoloured map where yellow indicates *p* < 0.05 and green indicates *p* ≥ 0.05. To further highlight only those regions for which the difference was clinically important, we repeated the analysis, counting only those cases that were growing faster than expected.

## Results

**Fig. 3:**
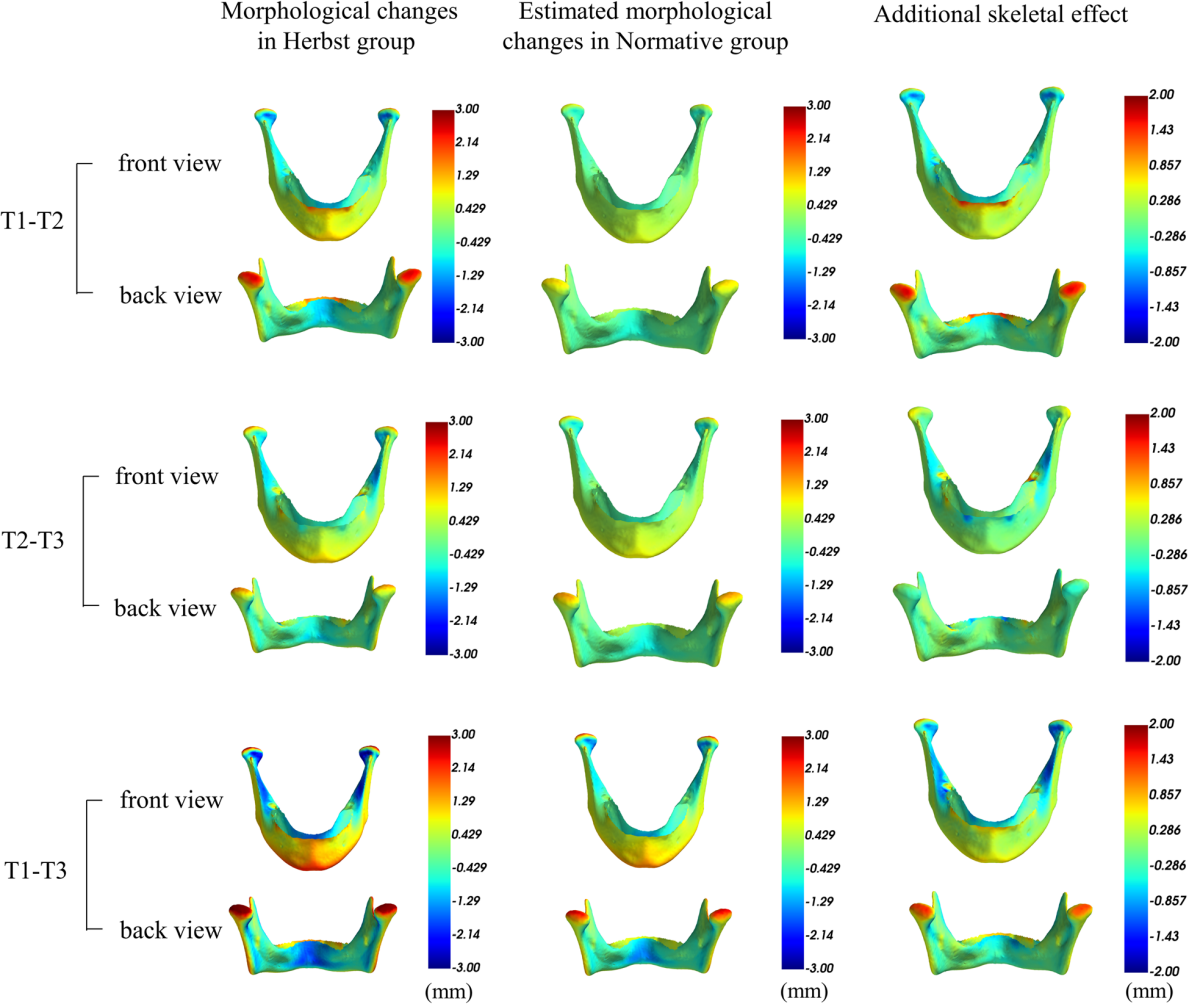
Additional skeletal effect produced by the Herbst appliance in 3D. This is calculated by contrasting the mean morphological changes in the first column to mean expected morphological changes due to natural growth in the second column from T1-T2, T2-T3 and T1-T3, respectively. The color maps indicate the amount of changes along the surface normals. Approximately 1.5–2 mm greater condylar changes (red) and 0.5 mm greater changes at the chin (yellow) are seen during active Herbst appliance treatment from T l to T2. This effect lasts until the completion of treatment (T1-T3), but there is no obvious skeletal effect during the orthodontic phase (T2-T3)

The mean mandibular morphological changes from T1 to T2 in the Herbst group were greatest for the condyles followed by the dento-alveolar bone, and the chin. Natural growth changes occurred in a similar manner, but to a lesser extent. From T1-T2, approximately 1.5–2 mm greater condylar change (increase in mandibular length), and 0.5 mm greater chin protrusion were observed. This effect persisted until the completion of treatment (T1-T3). There was no additional skeletal effect during the orthodontic phase of treatment (T2-T3) (Fig. 3).

**Fig. 4:**
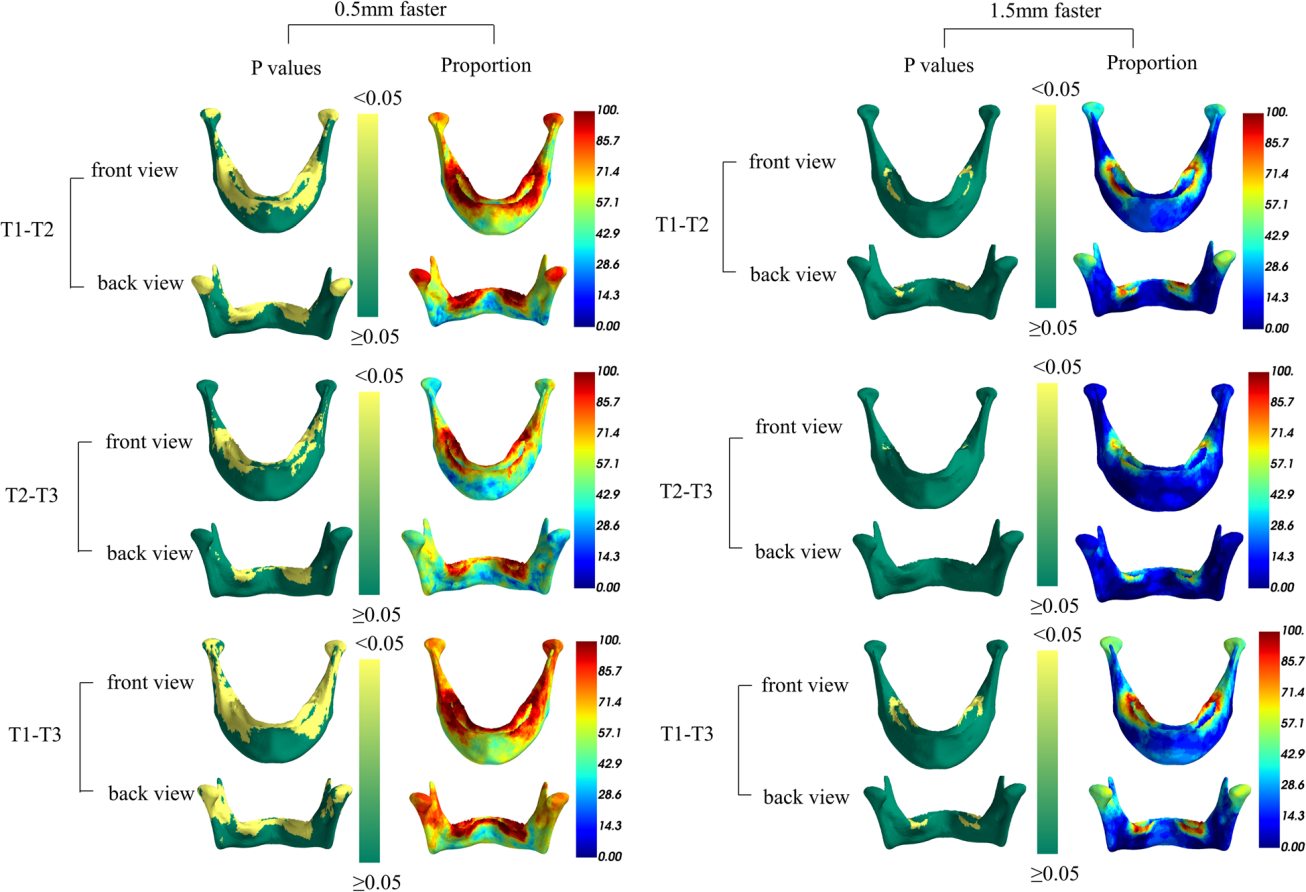
Statistical analysis of the additional skeletal effect. The left column indicates that approximately 85–100% of cases in the Herbst group have 0.5 mm additional condylar changes compared to the normative group from T1-T2 and that these changes are statistically significant. The right column indicates that only 40–50% of cases in the Herbst group have 1.5 mm additional condylar changes compared to the normative group from T1 to T2, and that these changes are not statistically significant. The additional changes at the chin are not statistically significant at both 0.5 mm and 1.5 mm cut-off values

Figure 4 shows the regions on the mandible with a statistically significant Herbst effect compared to the normative model (controls) at the cut-off values of 0.5 mm and 1.5 mm, and the proportion of the sample so affected. The left column documents that 85–100% of the Herbst group had a significant condylar change of > 0.5 mm increase in length compared to the normative group from T1-T2, which was reflected by the pointwise *P*-values under 0.05 at the condylar regions (in yellow). Furthermore, 40–50% of patients in the Herbst group had 1.5 mm more condylar change than the normative group from T1 to T2. Although the mean orthopedic changes at the condyles and the chin were more than normal growth (Fig. 3), they were not significant according to the binomial test because the variable effect was seen in less than half of the sample. In brief, a minority of patients with relatively large orthopedic effects failed to result in a significant difference for the entire sample.

## Discussion

The functional repositioning of the mandible with a Herbst appliance is usually directed at restoring a Class I occlusion with acceptable facial form. Optimal anterior posture allows for adaptive growth of the mandible to achieve the clinical objective, but it is not a primary mechanism forcing it to grow beyond normal growth potential [10]. Most malocclusions are manifestation of aberrant posture and/or pernicious functional habits. Mechanically repositioning the jaws with a fixed functional appliance to a near ideal sagittal and frontal relationship helps elicit catch-up growth to restore normal occlusion. In effect, a Herbst appliance eliminates the functional inhibition of growth, thereby allowing the jaws to assume a more normal occlusion via expression of inherent growth potential. The present study is consistent with this concept because none of the subjects had a history of true genetic malocclusions such as craniofacial anomalies or traumatic injury. Variable expression of an orthopedic effect exceeding the normal rate of growth (Figs. 2, 3 and 4) is expected because the patients had malocclusion associated with variable suppression of inherent growth potential. It is unlikely that any mechanical device can elicit mandibular growth beyond the inherent growth potential, but functional repositioning with a Herbst helps stimulate additional short-term growth [5].

To correct a skeletal malocclusion due to a functional inhibition of growth, it is necessary to achieve an orthopedic enhancement of mandibular length (condylar growth) that exceeds normal growth for untreated subjects. An orthopedic effect occurred in most of the patients, which was directly related to the degree of pretreatment suppression. The enhancement of growth did not exceed normal potential, but fixed functional treatment did provide a more optimal environment to achieve a fuller expression of it. This study has found that the growth response improved the occlusion and facial form by condylar and chin enhancement. Most corrections were within the range of normal growth, but 40–50% of the patients experienced additional dentofacial correction. Condylar growth of 1.5 mm or more was statistically significant (*p* < .05), but the small mean effect on the chin (0.5 mm) was not. These results confirm that a Herbst appliance has a variable effect on mandibular form (condyle and chin) that is directly related to the functional suppression of normal growth. Since a skeletal malocclusion is associated with variable amounts of functional suppression, a Herbst appliance is expected to be equally diverse orthopedic effect exceeding normal growth that depends on the specific etiology of a particular malocclusion.

The putative advantage of the Herbst appliance is producing an acceptable occlusal rehabilitation, while optimizing the skeletal outcome [9, 10]. Controversy continues about the possible influence of functional appliances on the basal skeleton of the jaws relative to outcomes from previous 2D studies [5]. 3D imaging modalities like CBCT provide a wealth of new data that is more than just an additional dimension. New tools and descriptive methods considerably exceed the capabilities of conventional cephalometrics. For instance, the emerging field of spatially-dense geometric morphometric analysis provides tools for the statistical analysis of the complete form of an object. In this regard, corresponding points were automatically applied all across the entire mandibular surface, which is less prone to error than manual identification of sparse landmarks. This approach allowed analysis of the whole surface of the mandible rather than projections of the mandibular contours in 2D images as described previously [20, 21]. Mandibles were then compared with robust Procrustes superimposition, which rotated and translated one mandible to optimally align it with another, giving greater weight in the alignment process to regions that are most similar to each other. This prevented a tendency for any change in form to subtly alter the superimposition. The color map plotted the differences between corresponding points of the mandible, which provided an intuitive visualization of the changes that related to the growth and treatment for clinicians. The dento-alveolar effect could also be quantified and visualized alongside morphological changes of the mandible as seen in Fig. 2.

As the morphological changes during each of the observation time periods contain both normal growth and changes due to treatment, the normal growth process that contributes to the correction must be factored out to evaluate the skeletal effect due to the appliance. Only two studies have evaluated in 3D the additional skeletal effects on the mandible by comparing patients treated with the device with control groups undergoing one phase non-orthopedic dental treatment [8, 22]. Like previous 2D studies, these have led to disparate conclusions in terms of the mandibular length, largely because obtaining a standardized control group is challenging in retrospective studies as it is hard to match the follow-up time precisely. One 2D study, by Lai and McNamara has contrasted cephalometric data in the Herbst group with population-based normative values derived from the University of Michigan Growth study over the same follow-up period [23]. They found a statistically significant increase in mandibular length in the active phase of the Herbst group compared with the normative group. The present study sought to utilize a similar method in 3D that focuses on changes at the condyles. The results agree with earlier conventional 2D studies that observed increases in mandibular length in patients treated with the Herbst appliance [9, 24–26]. The observed effect on condylar growth is also similar to that reported by Souki et al., who showed in 3D that the net growth of the condyles in all surfaces was significantly greater in the Herbst group [22]. The results indicate a true stimulation of bone apposition at the condyles, and ultimately may help maximize the skeletal outcome by generating substantially more growth in the sagittal dimension.

However, it appears that only a small number of cases in the Herbst group have more than 1.5 mm additional change at the condyles. Most cases have relatively small changes, which are unlikely to alter the form of the mandible in a clinically significant way. The correction of the molar relationship and overjet in these cases is likely to be due largely to dento-alveolar effects. Although both the skeletal effect on the mandible and the dento-alveolar effect could be visualized and quantified, it was not possible to ascertain whether variations in treatment effect were influenced by the skeletal maturity of the patients and timing of the treatment. The small sample size limited the degree of significance for the results. In addition, the skeletal effect has only been evaluated on a crowned and cantilevered Herbst-variant appliance and with a step-by-step advancement of the mandible. Herbst appliances based on variations in the anchorage units design, different vectors of intergnathic force exertion and different mandibular advancement protocols may have given a different outcome. With a larger sample of Herbst subjects, the methods described could be used to analyze this question in more detail.

A limitation of the study is that the normative group was derived from a clinical cohort which included skeletal Class I, II and III patients. A cohort specific to Class II individuals should be considered in the future to verify the skeletal effect of the Herbst. In addition, without matched longitudinal images of an appropriate control group, it is not possible to calculate the variation in growth rates on which to base a statistical inference on the effect of the appliance on rates of growth. The changes in the mandibles due to expected growth are based on cross-sectional data, and only estimate the central tendency. To circumvent this problem the assumption was made that growth rates in the population should be distributed symmetrically around this central tendency. This assumption allowed a limited statistical inference concerning the rate of change of the Herbst group compared with the normative group. Given the difficulties in gathering CBCT images from large numbers of untreated patients with a specific occlusion, such cross-sectional normative data currently provide the best available quantitative and statistical analysis of the additional skeletal effect produced by the Herbst appliance.

## Conclusions

The principal skeletal effect of Herbst appliance treatment was additional gain at the condyles, which contributes to increases in the sagittal dimension that aids in Class II correction. However, there is significant individual variation in the amount of changes in response to the Herbst appliance. Approximately 40–50% of the patient sample had > 1.5 mm increase in condylar length compared to growth of age and sex matched controls. Geometric morphometrics provides an efficient, intuitive and quantitative methodology for evaluating treatment effects that could be used for larger samples in the future.

## Data Availability

The datasets used during the current study are available from the corresponding author on reasonable request.

## Abbreviations

3D: three dimensional
CBCT: Cone-beam computed tomography
